# Development and Validation of the Socio-Ecological Scale for Identifying Delays in Treatment for Breast Cancer Patients

**DOI:** 10.1155/ijbc/2504583

**Published:** 2025-07-10

**Authors:** Sadia Jabeen, Rubeena Zakar, Florian Fischer

**Affiliations:** ^1^Department of Sociology, Virtual University of Pakistan, Lahore, Pakistan; ^2^Institute of Social and Cultural Studies, University of the Punjab, Lahore, Pakistan; ^3^Institute of Public Health, Charité-Universitätsmedizin Berlin, Berlin, Germany

**Keywords:** breast cancer, development, patients, scale, socio-ecological

## Abstract

**Background:** The study was aimed at measuring the factors contributing to the delayed presentation of breast cancer patients within the socio-ecological context by developing a scale.

**Methods:** The study objectives were measured by developing the items on the basis of a 5-point scale named the Socio-Ecological Scale for Breast Cancer Patients (SES-BCP). The dimensionality of the measure and internal consistency were determined by collecting data from 350 breast cancer patients from five main hospitals in three main cities (Lahore, Multan, and Faisalabad) in the Punjab province of Pakistan. A multistage sampling technique was employed, and sociodemographic factors were kept in consideration. Confirmatory factor analysis was applied for the factor structure in the study by using a structural equation model.

**Results:** With the distinctive five factors of the SES-BCP, a total of 51 items were confirmed in the final scale with sound psychometric properties, providing a multidimensional view of the study that helps in the early detection and cure of disease.

**Conclusions:** It can be concluded that this scale is a valuable addition to assess the underlying factors of delayed presentation in patients with breast cancer in the context of the socio-ecological model in Pakistan.

## 1. Introduction

At the global level, the most prevalent type of cancer is breast cancer. In 2022, as per the Global Cancer Observatory, about 20 million of cancer patients were diagnosed and 9.7 million deaths globally. Out of which 46.8% incidences of breast cancer were found with 12.7% deaths [1]. As per the International Agency for Research on Cancer (2018), about 15% of annual deaths occur in less developed countries as a result of breast cancer. It is one of the major public health issues that needs immediate action and attention, which could only be possible by identifying the underlying factors of the delay that are accelerating breast cancer patients' mortality. There are fewer chances of survival and prevention if there is a delay in treatment [2, 3]. The elapsed time between the first recognized symptom and diagnosis is often called “presentation delay.” A variety of definitions have been given by researchers previously to define the delay, as Yau et al. referred to it as the time span when a woman notices any change in the breast structure and consults the doctor to discuss these changes [4]. In the same vein, Corner et al. explained the delay of 3 months between the recognized symptom and diagnosis [5]. However, most of the researchers explained delay as the time between diagnosis and treatment [6–9]. The total delay is, however, considered the presentation delay; it includes two major dimensions, that is, the patient and the provider [10, 11]. A late consultation with the doctor, even after knowing the symptoms and ignoring them, is considered a patient delay. However, the provider delay is the time duration between first presentation, diagnosis, and treatment [12].

The delay is the result of many sociocultural factors that are very important to study [6, 13]. Therefore, identification and exploration of environmental, sociocultural, and community factors contributing to delay in the treatment of an individual are crucial. Thus, a multilevel parallel strategy is imperative to find out community, organizational, policy-level, interpersonal, and personal factors that directly influence the delay in treatment. In this regard, a socioecological approach to studying the presentation delay in breast cancer patients is being followed for this study. The delaying factors include poor socioeconomic conditions for women and gender-specific social and cultural factors that create hindrances to seeking medical care timely [14]. If diagnosed at an early stage, breast cancer treatment is possible [15]. However, individual, intrapersonal, community, organization, and policy factors are important to find out the actual causes of delay.

### 1.1. Intrapersonal/Individual Factors

Under the umbrella of socioecological framework, one of the factors contributing to the delay in treatment is lack of personal emphasis on their own health [16]. The late presentation of disease at the individual level was found to be mostly influenced by illiteracy and a lack of knowledge about the disease and its symptoms. The signs and effects of a disease's late identification and diagnosis are poorly understood [17]. Even literate females who are aware of the symptoms exhibit this tendency by delaying both self- and clinician-assisted assessment. Fear of mortality, hospital fear, fear of the unknown, and mastectomy are the four categories of fears related to breast cancer sickness that have been found by many researchers [18–20]. In Pakistan, breast patients have a poor survival rate and a higher mortality rate. Research indicates that different personalized variables, including ignorance, a lack of time brought on by domestic responsibilities, shock, and resistance, are the main barriers that Pakistani women reported as factors of delaying cancer screening [11]. The avoidance from screening process is also significantly influenced by other key personalized characteristics, which include denial, trouble remembering, shyness, and poor self-efficacy [21].

### 1.2. Interpersonal Factors

As per social perspective, a person's behavior, reactions, and actions are influenced by their beliefs, norms, values, culture, and social relationships. A patient's mental and physical health are influenced by their social network, which includes social influence and support, companionship, and social control [22]. Women with breast cancer in Pakistan have reported being reluctant to seek medical help for fear of being judged harshly by friends, family, coworkers, and people in their surroundings [23]. This problem often goes unnoticed, as major decisions for health services rest with male members who have financial autonomy and decision making power. Therefore, due to the strong influence of society, breast cancer patients willingly adhere to certain taboos and conditions [24–26].

### 1.3. Community Factors

To reduce health disparities, community factors are divided into four categories: environmental (such as housing or nutrition), social capital–based (such as social behavior), structural (such as ethnicity or economic factors), and service-based (such as literacy, public healthcare, and education). Socioeconomic determinants have an impact on cancer incidence at both the individual and social levels [27]. It has been found that a lack of basic cleanliness is one of the societal factors linked to both cancer and other diseases [28]. The cancer disease is also linked to various female sins and bad behaviors in traditional societies like India and Pakistan [29, 30]. Similarly, patients prefer to visit spiritual healers and traditional healers as a priority throughout Africa, Asia, and especially in South Asian nations as a common norm of the community [31]. The process of detection and presentation of breast cancer among patients is also being delayed by a lack of awareness campaigns at the community level [32]. Due to a lack of information, resources, distance from the medical facility, and a robust healthcare system, women in rural areas are more likely to continually face delays in the treatment of cancer [33].

### 1.4. Institutional Factors

Institutional help is defined as the way government or regulatory agencies provide and facilitate help to families and patients specifically. The tools, infrastructure, supplies, medications, staff, other financial resources, and medical healthcare system are institutional factors [32]. In Pakistan, it has been noted that patients look for institutional assistance at their places of employment for prompt disease treatment. By meeting their financial needs and fostering mental stability, it offers the patient and their families' tremendous support [33]. The health of patients is declining due to inadequate government support and insufficient levels of investment in the medical sector [34, 35]. Additionally, poor awareness, inadequate access to care, and inappropriate therapy utilization contribute to breast cancer patients' mortality [36, 37]. Unfortunately, due to a lack of institutional support from the government, the majority of institutes in Pakistan are not able to offer regular mammography and other screening tests that are necessary for early detection [38–40].

### 1.5. Policy-Level Factors

Efficient and effective policies can lead to rapid disease screening and management as well as public education to increase awareness of the significance of nutrition, weight control, and food in disease control [41]. Inadequate actions to spread awareness by government further increase the magnitude of the disease [42]. When breast cancer is diagnosed late, it brings with it a tangled web of factors that interact with one another and instill fear at every level of the socioecological system.

The existing studies focus on the different dimensions of breast cancer, such as one of the studies aimed at the socioeconomic status, race, ethnicity, and environmental exposures that cause delays in the treatment [43]. Later in 2001, another study conducted research on breast cancer in the context of environmental risk factors, genetics, and lifestyle as determinants of cancer [44]. Physical activity, diet, and environmental influences were further explored by Friedenreich et al. (2006) to determine the factors behind the cancer, particularly breast cancer [45]. The Breast Cancer Prevention Trial Lifestyle Questionnaire was developed in 2007 with a focus on dietary habits, hormonal factors, and physical activity in a large population with a special focus on cancer prevention [46]. Few studies focus on social capital, healthcare access, psychosocial stressors, and the role of this determinant in managing cancer [47–49]. Neighborhood environment, social support, and access to healthcare, as well as lifestyle, environmental exposures, and economic dependence, were elucidated to find the etiology of breast cancer [50, 51].

There is no empirical study that has been developed on the basis of the socioecological model based on the five factors, which is a way of addressing early detection, diagnosis, and treatment. The Socio-Ecological Scale for Breast Cancer Patients (SES-BCP) has the capacity to explore the causes of delay both in the developed and developing countries. This scale has an extensive scope as compared to existing research work, which is unidimensional; it provides a multidimensional view of the study that helps in the early detection and cure of disease.

The study had following objectives:

1. To develop an indigenous scale to measure socioecological factors of breast cancer delay
2. To determine the breast cancer scale's psychometric properties
3. To explore and highlight the importance of early detection on a socioecological scale as an innovative research area in the Pakistan context, where a significant number of women are diagnosed with disease at later stages

## 2. Materials and Methods

There were two phases followed to conduct this study. Item generation was done in Phase I of the study, while confirmatory factor analysis (CFA) was the focus of Phase II. For the purpose of achieving the theoretical structure of the scale, CFA was applied to empirically produce the items.

### 2.1. Phase I: Process of Item Generation

In three steps, the process of generation of items for SES-BCP was carried out: (i) an analysis of the factors of presentation delay on the basis of existing work or studies; (ii) finding out the experiences of breast cancer patients, five interviews were conducted in five different hospitals in five different cities of Punjab province; and (iii) expert opinion was taken on the group of items pooled after both steps. According to several studies, this strategy is generally accepted for creating concepts and scales for scale development [43, 44, 52–55].

A total of 60 items were obtained through this method and categorized according to socioecological framework levels. Being an expert in the field of public health, the study supervisor further examined the items using the following four criteria: (i) content clarity, (ii) reading clarity, (iii) reiteration, and (iv) construct relevance. For pretesting, 55 items were finally selected. On a 5-point scale, each item was assigned a category: 5 for *strongly agree*, 4 for *agree*, 3 for *undecided*, 2 for *disagree*, and 1 for *strongly disagree*. The following three measures were used to ensure the psychometric screening of the items: (i) each statement's comprehension and clarity, (ii) ensuring the questionnaire's length and limit, and (iii) the suitability of the scale's components.

Table 1 presents an overview of the factors, operational definitions, and sample items. All these factors were developed on 5-point Likert scale (*strongly agree* = 5, *agree* = 4, *neutral* = 3, *disagree* = 2, and *strongly disagree* = 1).

### 2.2. Phase II: Internal Consistency of Scale and Factorial Structure

A total of 50 respondents participated in the initial scale's pilot testing, 10 from each hospital and the data was collected physically through face-to-face interaction. Based on their responses, four questions were eliminated from the scale because they were repetitive, and five items were rephrased to make them more understandable. In order to evaluate the discriminant validity, convergent validity, and reliability of the factors, a CFA was run on a 51-item scale. The questionnaire was launched fully after a reliability analysis of pilot testing. With a Cronbach's alpha value greater than 0.8, the scale was determined to be reliable.

### 2.3. Study Participants

Patients with breast cancer were the study's respondents. The method of multistage sampling was employed to choose the respondents. The researcher created the strata for the selection of the respondents based on the available demographic and hospital information in the cities of Punjab province, Faisalabad, Multan, and Lahore, where the oncology wards were found in five public sector hospitals. Three hospitals were chosen from the Lahore Division, one from the Faisalabad Division, and one from the Multan Division. A total of 350 respondents were selected, 70 from each institution. The respondents' average age was 43.4 years. Almost half of respondents (49.7%, *n* = 174) were not educated; 9.7% had education until grade five, and 10.6% had education until grade eight. About 5.1% of respondents had 12 years of schooling, 5.4% had 14 years of education, and 5.4% had postgraduate-level education, compared to about 15% of respondents who had schooling up to the tenth grade. The majority of respondents (79.7%, *n* = 279) were married, followed by 10.6% of singles, 8.9% of widows, and 1% of divorced.

### 2.4. Data Collection

A total of 51 items based on the SES-BCP were finalized after pilot testing.

According to the definition used in this study, a patient's presentation was considered delayed if it took longer than 3 months. If it took less than 3 months, it was not considered to be delayed.

In order to collect the data from the hospitals, the hospital's administration and medical superintendents were asked for their approval. In this regard, the researcher physically visited each hospital, made an appointment in advance with each medical superintendent, and explained the goal of the research in a letter that had been signed by the director of the Institute of Social and Cultural Studies and the supervisor. Additionally, the researcher provided the tools for data collection when requested and a copy of the research proposal that had been ethically approved and accepted by the Board of Advance Studies and Research (D/8555/Acad.) as a part of PhD research. Additionally, the researcher gave the authorities assurances regarding the privacy and anonymity of the data. Each participant was given a personalized description of the study's goals, and from the willing participants, informed consent was also sought in the form of a signed document. The respondents were also guided through the five categories of responses on the scale, and they were instructed to select the best option. Although the scale was created in Urdu as well, the researcher herself recorded the response by explaining the question to the illiterate subjects. Names and other personally identifiable information were not included in the questionnaire to maintain the anonymity and confidentiality of the data.

## 3. Results

**Figure 1:**
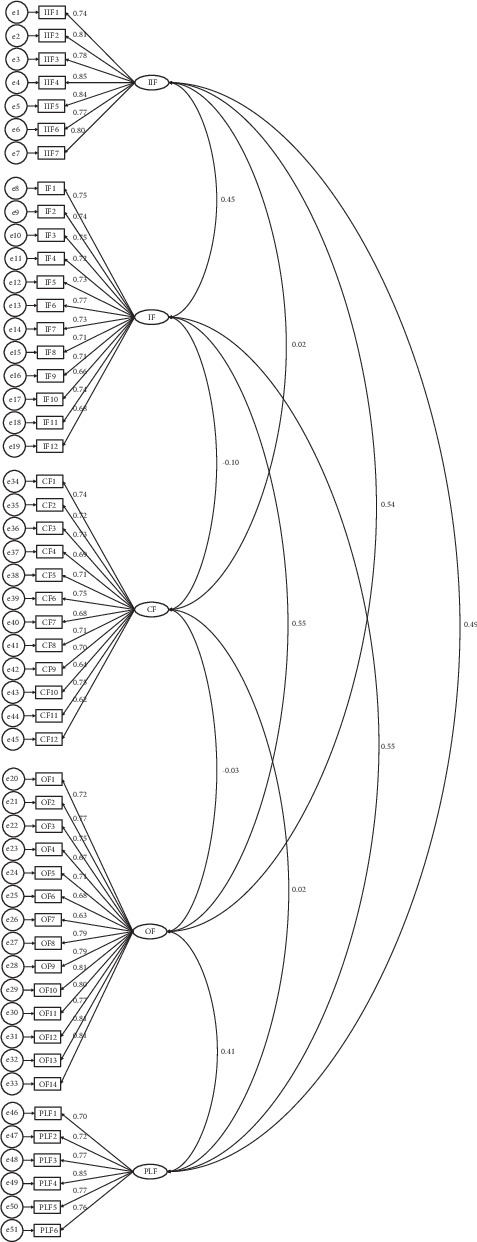
First-order confirmatory factor analysis for Socio-Ecological Scale for Breast Cancer Patients (SES-BCP).

The factorial structure of the SES-BCP was validated through CFA with a 5-point scale on 51 items. AMOS (analysis of moment structure) through CFA (Figure 1) was applied to the SES-BCP by using the IBM SPSS 25.0 Version. Policy-level, organizational, community, interpersonal, and individual/intrapersonal factors were the five subscales included in the socioecological scale for identifying the delay in treatment for breast cancer patients. The model fit indices are listed in Table 2.

The absolute model of CFA was 2 (1214) = 2152.55, *p* < 0.05, which was the model fit for the breast cancer patients' socioecological scale. A great fit from the analysis of the absolute model demonstrated that population variance-covariance and sample variance-covariance were both equal. However, it is believed that the sample size and number of parameters that must be evaluated in the conventional model are important factors in the chi-square test. In light of this statistical oddity, statistical science theorists advised using a variety of indices to quantify model fit, also known as the relative fit of the model. Hence, to measure the consistency between the tested model and data, the root mean square error of approximation (RMSEA), the goodness-of-fit index (GFI), the nonformed fit index (NNFI) via Tucker–Lewis fit index (TLI), the standardized root mean square error of approximation (SRMR), and the cumulative fit index (CFI) were calculated.

Several statisticians have provided thresholds to determine whether the indices were appropriate for model fit [45, 46]. These threshold requirements specify that the RMSEA and SRMR indices must be less than 0.08 and 0.05, respectively—lesser is preferred—and that the 22/*df* should be between 0 and 3. Although 0.90 or higher is required for the null model comparison, 0.80 is occasionally recognized as the standard for the GFI, CFI, and TLI indices.

Due to this, it was determined via an analysis of the fit of the current model that the RMSEA and SRMR were, respectively, 0.04 and 0.05. While the chi-square to degree of freedom (2/*df*) was determined to be 1.77, the analysis of the comparison of the null models, that is, GFI, CFI, and NNFI for (SES-BCP), was 0.94, 0.92, and 0.91, respectively. As a result, it is evident that the initial test model complies with all model fit recommendations. Therefore, it may be concluded that the population variance-covariance and sample variance-covariance are consistent with the data.

To determine the psychometric properties of the socioeconomic scale for breast cancer patients, including convergent and discriminant validity and reliability following the completion of the model fit, the results of the CFA were evaluated. Research suggests that Cronbach's alpha reliability and reliability coefficients for composite reliability should be greater than 0.70. In the same vein, the average variance extracted (AVE) index must be a minimum of 0.50 or greater [46, 47].

The average of the variances for each item of the factors was assessed to determine the convergent validity as a result of the analysis of the factor loading of the items of the scale (Table 3). Each component was found to have a factor loading of at least 0.70, meaning that the associated variance for each individual item was at least 0.50 [45]. However, it was found that the SES-BCP satisfied the strict requirements of convergent validity. Individual/interpersonal, interpersonal, organizational, community, and policy-level variances each explained an adequate percentage of the SES-BCP, that is, 64, 52, 59, 50, and 58, respectively. Patients with breast cancer had composite reliability coefficients of the factors that varied from 0.89 to 0.95 at the same time.

Two alternative methods were used to establish the discriminant validity [47, 48]. The correlations of the factors in the first technique (Table 4) were compared with the square root of the average variance recovered from each element's AVE ratio [49]. Thus, the square root of AVE is greater than the correlation as found in the results. Each factor's individual AVE and maximum shared variance (MSV) were assessed through the second method. The percentage of the same component's explained variance should be larger than that of any other factor, and the value of the average variances retrieved should be higher than the MSV. Since the average variance retrieved exceeded the sum of the shared variances of all important components, it was clear from the estimations that this was higher in this case [45].

The socioecological aspects (individual/intrapersonal, interpersonal, organizational, community, and policy) of breast cancer patients were also documented using Cronbach's alpha. The Cronbach's alpha is in the excellent range of internal consistency, falling between 0.89 and 0.95 (Table 5).

Pearson product–moment correlation was performed to determine the inter-item correlation between sociological factors, that is, interpersonal/intrapersonal, interpersonal, organizational, community, policy level, and delay in the treatment of breast cancer patients. Results showed that there was a significant positive correlation (*p* < 0.001) between individual/intrapersonal, interpersonal, organizational, and community factors and delay in the treatment in patients having breast cancer. Results were also found significant between interpersonal factors, organizational factors, community factors, and delay in treating breast cancer. Interpersonal factors were negatively correlated (*p* < 0.001) with policy-level factors, whereas policy-level factors were also significantly inversely correlated (*p* < 0.001) with organizational factors. A single scale score may provide a holistic measure that reflects the combined impact of multiple factors on the outcome of interest. Even if certain factors are negatively correlated, they may still contribute meaningfully to the overall construct being measured. For example, in organizational or policy research, the tension between different levels (interpersonal, organizational, and policy) is often inherent but still part of the broader framework. Moreover, sociological factors (policy level and community) were positively correlated with delay in the treatment of breast cancer patients, as well as the significant positive correlation between organizational factors, community factors, and delay in treatment of breast cancer patients was found. The results indicate a significant positive association (*r* = 0.750, *p* < 0.001) between treatment delay and community variables, indicating that higher treatment delay scores are linked to higher community factor scores. This correlation has a substantial effect size and is statistically significant (Table 6).

## 4. Discussion

In this paper, the SES-BCP is used to measure individual, interpersonal, social, cultural, environmental, organizational, and policy factors by using CFA. The purpose of the study was to find out the underlying factors causing delay in the treatment of breast cancer patients by identifying and analyzing socioecological factors in an indigenous cultural context. Various studies had tried to find out the factors contributing to the delay in the treatment of breast cancer patients but were unable to follow a holistic approach to study all the factors contributing to the delay. The phenomenon of delay remained undermeasured due to focusing only on one or two side factors, which did not give a true depiction of the issue previously. A socioecological approach is a comprehensive way to understand the personal, interpersonal, community-based, organizational, and policy-level issues involved in the delayed presentation of patients to healthcare facilities.

The authors of the study adopted a socioecological model to measure the delay; in this regard, extensive literature reviews and qualitative interviews were conducted to carry out the item generation process. The socioecological factors involved in the delay were addressed in this study. This study provides a holistic measure to identify the factors causing delay in the treatment of breast cancer patients in Pakistan.

The study's findings were in line with earlier studies in this area that claimed that middle- and low-income countries experience delayed presentation [50]. Additionally, it was suggested by study results that socioecological elements (individual/intrapersonal, interpersonal, organizational, community, and policy) were discovered to be considerably positively linked with overall delays. The findings of the study were confirmed by earlier investigations describing the relationship between elements of the healthcare system and effective disease treatment [51]. However, because they focused on single-level characteristics, earlier studies were unable to offer adequate support in this situation. However, neither of the two types of presentation delays included the sociocultural or ecological environment, which may have delayed diagnosis or treatment. The sociocultural background of the delay in breast cancer patients' treatment has received little scientific attention [56–58]. According to Unger-Saldaa and Infante-Castaeda [10], the sorts of delays described in earlier studies neglected the whole picture, handled patient and provider delays separately, and disregarded interactions and connections at several levels that were implicated in the actual delays. This study took a more practical approach, recognizing that delay is not the result of a discrete or singular source [35].

The results of recent research even showed that screening services in hospitals at the time were insufficient. The patient's therapy was further delayed by inadequate equipment and treatment mechanisms. Furthermore, a sizable population suffers more from going to these hospitals because the available facilities are exclusively limited to the hospitals in the major cities. Treatment becomes more challenging for families living in poverty when those families live far from tertiary hospitals or other healthcare facilities. Females living in remote places and villages had to pay for their trip expenses as well as incomplete information about the registration and screening processes due to the lack of specialized physicians and health centers. The patient had to wait a long time before entering the process. Additionally, the difficulty of finding an accompanying person who lives far away, the time it takes to get to the medical facility, and the operating hours of the indoor and outdoor units prevent patients from visiting hospitals on a regular basis [59–61]. Among the important components of the socioecological model that affect patients' attitudes about illness treatment and other health-seeking behaviors are community-level factors. In addition, the physical and social surroundings in which people go about their everyday lives, their patterns of eating and physical exercise, and their daily routines all influence the condition. At the community level, other factors include health literacy, awareness, and the availability of services to help fight the illness. The local population has an impact on the networks of social support systems that include family, peers, and organizations [62, 63].

The study has certain limitations. The study was cross-sectional in nature, so more research with a larger sample size is needed for generalization of findings. The data for this study was collected from public sector hospitals only; further research can be conducted in private sector hospitals as well. Moreover, the study is limited to the Punjab province only; future studies can be conducted in other provinces as well.

## 5. Conclusion

On the basis of the findings of the study, it can be concluded that the SES-BCP is a reliable and valid scale to measure the delay in the treatment of breast cancer patients in Pakistan. The widespread categories of measuring the socioecological factors of delay under the five main factors of the socioecological framework in the indigenous context provide a new measure that has sound reliability and validity as well as psychometric properties. So, it can be concluded that the SES-BCP is a promising scale with adequate homogeneity of items, accepted validity, and significant internal consistency.

## Figures and Tables

**Table 1:** Factors, operational definitions, and sample items.

**Factor**	**Operational definition**	**Sample items**
Intrapersonal factors	“The characteristics of the individual such as knowledge, attitudes, beliefs, behavior, self-concept, skills, etc.”	• The basic signs and symptoms of breast cancer were known to me before the diagnoses. • I was fearful of treatment. • I could not afford treatment so my treatment was delayed.

Interpersonal factors	“Primary groups—formal and informal social network and social support systems, like family, work group, and friendship networks.”	• When I tell the family about my health issue, they encourage me to seek medical help. • On the basis of family and friend's discussion about treatment I avoid to consult doctor. • Mostly family and friends associated it with some sin that I might have done in the past. • The decision of my treatment depends on the male members of the family (father, brother, and husbands)

Community factors	“Interactions among organizations, institutions, and informal networks within defined boundaries.”	• In our area it is not a tradition to consult doctors due to purdah. • People prefer spiritual healing in case of serious diseases. • Hospital is far away from my residence that it delays my decision to start treatment. • Different representatives of organizations come to provide awareness.

Institutional factors	“Social institutions with administrative characteristics, and formal (and informal) rules and regulations for operation.”	• I face difficulty in getting appointment to see the doctor. • I had to wait for a long time to get the test results from hospital. • Guidance from hospital is sufficient for patient. • I am satisfied with my treatment.

Policy-level factors	“Local, state, and national laws and policies. It is an assumption that these levels of analysis in health promotion interventions are based on our beliefs, understandings, and theories of the determinants of behavior.	• The awareness campaign being run to create awareness is sufficient. • Government promotes breast feeding practice for mothers to avoid the disease. • The socio-cultural aspect to treat the patients is considered.

**Table 2:** Fit indices of confirmatory factor analysis for the Socio-Ecological Scale for Breast Cancer Patients (SES-BCP).

	**χ** ^2^	**d** **f**	**χ** ^2^/**df**	**GFI**	**CFI**	**NNFI**	**RMSEA**	**SRMR**
Model fit	2152.55	1214	1.77	0.94	0.92	0.91	0.04	0.05

*Note*: *N* = 350. Relative to the model, *χ*^2^ > 0.05 all changes in chi-square values are computed.

Abbreviations: CFI, comparative fit index; NNFI, nonnormed fit index; RMSEA, root mean square error of approximation; SRMR, standardized root mean square.

**Table 3:** First-order confirmatory factor analysis for the Socio-Ecological Scale for Breast Cancer Patients (SES-BCP).

**Factors**	**CR**	**AVE**	**MSV**	**λ**
Individual/interpersonal factors	0.925	0.64	0.289	
IIF1				0.745
IIF2				0.811
IIF3				0.782
IIF4				0.850
IIF5				0.836
IIF6				0.767
IIF7				0.798
Interpersonal factors	0.929	0.52	0.308	
IF1				0.748
IF2				0.738
IF3				0.748
IF4				0.715
IF5				0.726
IF6				0.770
IF7				0.730
IF8				0.712
IF9				0.705
IF10				0.661
IF11				0.740
IF12				0.681
Organizational factors	0.952	0.59	0.308	
OF1				0.717
OF2				0.771
OF3				0.746
OF4				0.674
OF5				0.708
OF6				0.675
OF7				0.828
OF8				0.789
OF9				0.789
OF10				0.808
OF11				0.796
OF12				0.770
OF13				0.810
OF14				0.805
Community factors	0.922	0.50	0.010	
CF1				0.737
CF2				0.721
CF3				0.728
CF4				0.687
CF5				0.713
CF6				0.749
CF7				0.679
CF8				0.708
CF9				0.700
CF10				0.641
CF11				0.753
CF12				0.625
Policy-level factors	0.892	0.58	0.307	
PLF1				0.696
PLF2				0.723
PLF3				0.771
PLF4				0.853
PLF5				0.765
PLF6				0.750

*Note*: *λ* (lambda), standardized factor loading ≥ 0.4.

Abbreviations: AVE, average variance extracted; CR, composite reliability; MSV, maximum shared variance.

**Table 4:** Descriptive statistics and Fornell–Larcker criterion for the factors of the Socio-Ecological Scale for Breast Cancer Patients (SES-BCP).

**Factors**	**K**	**M** (**SD**)	***PF***	**IIF**	**IF**	**OF**	**CF**
PLF	6	21.26 (5.92)	0.761				
IIF	7	20.34 (8.45)	0.494	0.799			
IF	12	44.32 (10.65)	0.554	0.451	0.723		
OF	14	47.88 (14.08)	0.406	0.538	0.555	0.765	
CF	12	44.52 (10.06)	0.024	0.023	-0.102	-0.034	0.704

Abbreviations: CF, community; IF, interpersonal; IIF, individual/intrapersonal; *k*, number of items; *M*, mean; OF, organizational; PLF, policy; SD, standard deviation.

**Table 5:** Reliability coefficients and descriptive statistics for individual/intrapersonal, interpersonal, organizational, community, and policy factors in women with breast cancer (*N* = 350).

**Variables**	**K**	**M**	**S** **D**	**Range**		**α**
				**Actual**	**Potential**	
Individual/intrapersonal factors	7	20.34	8.45	7–35	7–35	0.93
Interpersonal factors	12	44.32	10.65	14–59	12–60	0.93
Policy factors	6	21.26	5.92	6–30	6–30	0.89
Organizational factors	14	47.88	14.08	14–70	14–70	0.95
Community factors	12	44.52	10.06	14–60	12–60	0.92

Abbreviations: *α*, Cronbach's alpha; *k*, number of items; *M*, mean, SD, standard deviation.

**Table 6:** Inter-item correlation of socio-ecological factors (individual/interpersonal, interpersonal, organizational, community, and policy) and delay in treatment (*N* = 350).

**Variables**	**2**	**3**	**4**	**5**	**6**
1. Individual/intrapersonal	0.429⁣^∗∗∗^	0.021	0.516⁣^∗∗∗^	0.449⁣^∗∗∗^	0.308⁣^∗∗∗^
2. Interpersonal		−0.098	0.537⁣^∗∗∗^	0.504⁣^∗∗∗^	0.307⁣^∗∗∗^
3. Policy			−0.029	0.019	0.207⁣^∗∗∗^
4. Organizational				0.376⁣^∗∗∗^	0.329⁣^∗∗∗^
5. Community					0.358⁣^∗∗∗^
6. Delay in the treatment					0.750⁣^∗∗∗^

⁣^∗^*p* < 0.05, ⁣^∗∗^*p* < 0.01, and ⁣^∗∗∗^*p* < 0.001.

## Data Availability

The data that support the findings of this study are available on request from the corresponding author. The data are not publicly available due to privacy or ethical restrictions.

## Nomenclature

AMOS: analysis of moment structure
AVE: average variance extracted
CFA: confirmatory factor analysis
CFI: cumulative fit index
GFI: goodness-of-fit index
MSV: maximum shared variance
NNFI: nonformed fit index
RMSEA: root mean square error of approximation
SES-BCP: Socio-Ecological Scale for Breast Cancer Patients
SRMR: standardized root mean square error of approximation
TLI: Tucker–Lewis fit index
